# Tipping the MYC–MIZ1 balance: targeting the HUWE1 ubiquitin ligase selectively blocks MYC-activated genes

**DOI:** 10.15252/emmm.201404735

**Published:** 2014-11-03

**Authors:** Franz X Schaub, John L Cleveland

**Affiliations:** 1 Department of Tumor Biology, The Moffitt Cancer Center and Research InstituteTampa, FL, USAJohn.Cleveland@moffitt.org DOI 10.15252/emmm.201404735 | Published online 3 November 2014

## Abstract

MYC family oncoproteins (MYC, N-MYC and L-MYC) function as basic helix-loop-helix-leucine zipper (bHLH-Zip) transcription factors that are activated (i.e., overexpressed) in well over half of all human malignancies (Boxer & Dang, 2001; Beroukhim *et al*, 2010). In this issue of *EMBO Molecular Medicine*, Eilers and colleagues (Peter *et al*, 2014) describe a novel approach to disable MYC, whereby inhibition of the ubiquitin ligase HUWE1 stabilizes MIZ1 and leads to the selective repression of MYC-activated target genes.

See also: **S Peter *et al*** (December 2014)

Targeting MYC is a high priority for cancer therapeutics. This is no easy task, as transcription factors are notoriously difficult to inhibit with small molecules, and selectivity is also a hurdle, as MYC oncoproteins heterodimerize with MAX, a requisite and related bHLH-Zip protein that dimerizes with other bHLH-Zip proteins (Blackwood & Eisenman, 1991). Finally, achieving a suitable therapeutic window is also a concern, as MYC proteins are required for development and for the growth of normal cell types, where the MYC:MAX complex binds to and directly induces or represses the transcription of a large cast of target genes that harbor CAC/AGTG E-box elements, which in turn then provoke a proliferative state that amplifies global RNA production (Lin *et al*, 2012; Nie *et al*, 2012; Sabo *et al*, 2014; Walz *et al*, 2014). Regardless, hope for targeting MYC has recently come from the development of compounds that disrupt the MYC–MAX interaction (Hart *et al*, 2014), that provoke MYC destruction (Brockmann *et al*, 2013) or that block the transcription of *MYC* itself, by targeting the bromodomain protein BRD4 (Delmore *et al*, 2011). Eilers and colleagues (Peter *et al*, 2014) attempt novel approach to disable MYC, by inhibiting the ubiquitin ligase HUWE1 to stabilize MIZ1, thus leading to the selective repression of MYC-activated target genes.

Over the past 2 years, the concept that MYC oncoproteins function as selective transcription factors was challenged by studies, suggesting that MYC was a non-specific amplifier of all active genes (Lin *et al*, 2012; Nie *et al*, 2012). More recent findings from the Amati and Eilers laboratories have, however, shown that: (i) transcription regulation by MYC is indeed selective; (ii) transcriptional amplification reflects a secondary response to the proliferative state that is provoked by the expression of direct MYC targets; and (iii) the ratio of MYC and MIZ1 bound to each promoter controls if a given target gene is activated or repressed (Sabo *et al*, 2014; Walz *et al*, 2014). Peter *et al* (2014) now exploit this MYC-MIZ1 balancing act, where they show that inhibiting the HUWE1 ubiquitin ligase in colon cancer cells tips the response in favor of MIZ1.

Heretofore, HUWE1 was known to function as an E3 ligase that ubiquitylates and directs the destruction of N-MYC and MIZ1 (Zhao *et al*, 2009; Yang *et al*, 2010) and to add K63-linked ubiquitin chains to MYC without affecting its turnover (Adhikary *et al*, 2005). Based on the newly recognized importance of the MYC to MIZ1 ratio at a given promoter, Peter *et al* reasoned that HUWEI1 might be a target that could be exploited to override MYC transcriptional programs. Specifically, the authors hypothesized that blocking HUWE1 expression or function would stabilize MIZ1 and lead to binding of MIZ1 to MYC:MAX complexes at key target genes, to switch transcription into an off state and disable cancer cell growth.

The authors used an array of approaches to test this hypothesis. First, as predicted, knockdown of HUWE1 effectively blocked colorectal cancer cell growth *ex vivo* and, importantly, blocked the progression of tumor xenografts *in vivo*. These responses were not due to direct effects on MYC levels but were rather squarely pinned on MYC transactivation functions, where a large cast of genes normally induced by MYC was now repressed. Further, the effects of HUWE1 depletion on MYC-activated genes were not due to indirect effects on cell cycle arrest, as many of these targets are not cell cycle regulated, and they were also independent of possible effects on the p53 circuit. Most intriguingly, the effects of HUWE1 knockdown on MYC-activated genes were selective, as there were no effects of genes normally repressed by MYC.

These findings motivated an *in vitro* ubiquitin-based screen of a large library of compounds (> 840K), to identify small-molecule probes that selectively blocked the auto-ubiquitination of the HECT domain by HUWE1 in the presence of the E1 UBA1 and the E2 UbcH5b. Top hits from the screen were then counter-screened for activity against UBA1, UbcH5b and the ubiquitin ligase NEDD4, and the top two passing muster were shown to block the ubiquitination of validated targets of HUWE1 in cells, including that of the anti-apoptotic protein MCL1 and the checkpoint protein TopBP1. Notably, the genetic studies provided suggest that the top two hits identified, which have rather modest potency (IC_50_ of 0.9–3 μM), do indeed target HUWE1. Most importantly, treatment of colorectal cancer cells with these agents, but not treatment of normal colonic epithelial cells or embryonic stem cells, triggered cell growth arrest and, again, blocked the expression of target genes that are activated by MYC, without affecting those that are repressed by MYC. Finally, the HUWE1 inhibitors had little-to-no effects on MYC target genes expression in cells already depleted of HUWE1.

Proof of the relevance to the HUWE1-to-MIZ1 circuit came from a series of convincing studies that established that: (i) inhibition or knockdown of HUWE1 induced stabilization of MIZ1 and triggered MIZ1 binding on target genes normally activated by MYC; (ii) inhibition of HUWE1 has no effect on the formation of MYC:MAX complexes nor upon the expression of MXD proteins that also dimerize with MAX; and (iii) knockdown of MIZ1 reversed most of the effects of HUWE1 inhibition or silencing.

Collectively, these findings suggest that MYC can selectively be targeted in cancer by disabling the HUWE1 ubiquitin ligase that normally controls MIZ1 protein levels (Fig1). In tumors where there is a preponderance of MYC oncoproteins, the balance is in favor of transcription activating MYC:MAX complexes, which induce the expression of their direct targets that then in turn provoke a hyperproliferative state that amplifies transcription. Inhibition of HUWE1 and elevated levels of MIZ1 then restores this balance, as MIZ1 binds to MYC:MAX complexes to form ternary MIZ1:MYC:MAX complexes that repress genes that are activated by MYC, thus abolishing the hyperproliferative response (Fig1).

**Figure 1:**
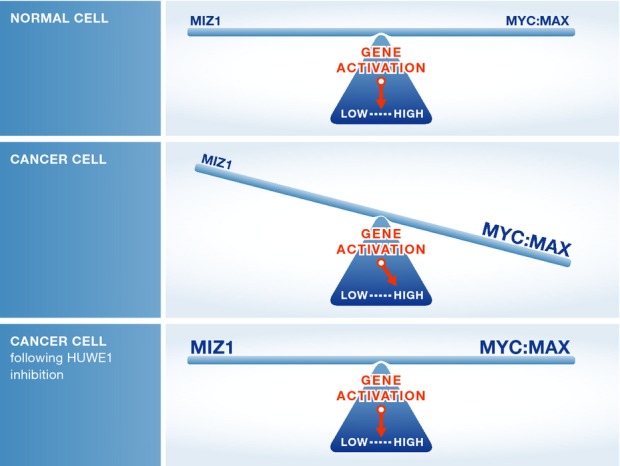
MIZ1–MYC equilibrium controls cell fate In normal cells, HUWE1-directed ubiquitylation of MIZ1 controls its levels to balance the control of MYC transcription targets. In cancer, MYC oncoproteins are overexpressed, which tips the balance to activating MYC:MAX complexes that activate direct targets, which in turn lead to a hyperproliferative state that includes an amplification of transcription (Lin *et al*, 2012; Nie *et al*, 2012; Sabo *et al*, 2014; Walz *et al*, 2014). Inhibition or silencing of HUWE1 in cancer cells re-establishes a proper equilibrium, by provoking increases in the levels of MIZ1 that forms repressive MIZ1:MYC:MAX ternary complexes on growth-associated target genes that are activated by MYC.

The small molecules identified thus far are, however, only the first steps toward targeting this circuit in the oncology clinic. Indeed, the current tool compounds lack the potency and proper drug-like properties needed for *in vivo* testing of safety and efficacy. Moreover, once developed, such HUWE1-targeting agents may have to be used in combination with other drugs, as knockdown of HUWE1 alone is not sufficient to induce tumor regression. Finally, other important studies need to be performed before attempting to translate these findings and include those confirming the role of this circuit in additional MYC-driven malignancies and those that interrogate possible mechanisms of resistance to such agents, which, for example, could include silencing of MIZ1 or gain-of-function somatic mutations in *HUWE1* that block the function of these small molecules. Nonetheless, the facts that HUWE1 is synthetically lethal for MYC-expressing tumor cells and that this is a tractable enzyme amenable to therapeutics raises hope that drugs that target this ubiquitin ligase can ultimately be added to our armament of agents to treat the broad spectrum of aggressive malignancies that have *MYC* involvement.
